# Deep learning for identifying bee species from images of wings and pinned specimens

**DOI:** 10.1371/journal.pone.0303383

**Published:** 2024-05-28

**Authors:** Brian J. Spiesman, Claudio Gratton, Elena Gratton, Heather Hines

**Affiliations:** 1 Department of Entomology, Kansas State University, Manhattan, Kansas, United States of America; 2 Department of Entomology, University of Wisconsin–Madison, Madison, Wisconsin, United States of America; 3 Department of Entomology, University of Illinois Urbana-Champaign, Champaign, Illinois, United States of America; 4 Department of Entomology, Penn State University, State College, Pennsylvania, United States of America; University College London, UNITED KINGDOM

## Abstract

One of the most challenging aspects of bee ecology and conservation is species-level identification, which is costly, time consuming, and requires taxonomic expertise. Recent advances in the application of deep learning and computer vision have shown promise for identifying large bumble bee (*Bombus*) species. However, most bees, such as sweat bees in the genus *Lasioglossum*, are much smaller and can be difficult, even for trained taxonomists, to identify. For this reason, the great majority of bees are poorly represented in the crowdsourced image datasets often used to train computer vision models. But even larger bees, such as bumble bees from the *B*. *vagans* complex, can be difficult to separate morphologically. Using images of specimens from our research collections, we assessed how deep learning classification models perform on these more challenging taxa, qualitatively comparing models trained on images of whole pinned specimens or on images of bee forewings. The pinned specimen and wing image datasets represent 20 and 18 species from 6 and 4 genera, respectively, and were used to train the EfficientNetV2L convolutional neural network. Mean test precision was 94.9% and 98.1% for pinned and wing images respectively. Results show that computer vision holds great promise for classifying smaller, more difficult to identify bees that are poorly represented in crowdsourced datasets. Images from research and museum collections will be valuable for expanding classification models to include additional species, which will be essential for large scale conservation monitoring efforts.

## Introduction

One of the most challenging aspects of bee ecology and conservation is classifying or identifying individuals by species. With more than 20,000 species globally, researchers may find tens to several hundred bee species to identify in any given study [e.g., 1–3]. The identification process requires a high level of taxonomic expertise because many species share similar and sometimes highly variable features. Such expert services are in decline [4] for both researchers and for the general public, who are increasingly providing crowdsourced data used to assess large scale trends in biodiversity [e.g., 5,6]. Computer vision is a promising approach that will help address the problem of bee species identification, especially for the larger taxa, such as *Bombus*, for which sufficient and accurate crowdsourced training datasets exist [7–9]. However, the effectiveness of computer vision for identifying smaller, more taxonomically challenging species is not well tested.

Computer vision can be effective for species-level bee identification, but this requires large, annotated image datasets for model training. Public occurrence and image repositories, such as the Global Biodiversity Information Facility (GBIF; https://www.gbif.org), provide access to images from citizen science programs and museums. For example, GBIF is an excellent resource for many species in the genus *Bombus* and other species that can be readily identified by experts from photos. However, most bee species are much smaller than *Bombus* with distinguishing features that are often obscured or not sufficiently resolved in photos for identification, even by experts. One such group of bees belongs to the subgenus *Lasioglossum* (*Dialictus*), which are particularly challenging to identify [10]. In North America, where they comprise over 250 species, *Dialictus* species are frequently encountered in scientific studies, yet they are often not identified to species because there are so few experts available [11]. Even DNA barcoding often fails for this "nightmare taxon" [12].

But even larger bumble bees can be difficult to identify without close inspection. For example, species in the *Bombus vagans* mimicry complex (*B*. *vagans*, *B*. *sandersoni*, and *B*. *perplexus*) have similar patterns of hair color and are frequently confused. Although they can be separated by using DNA barcodes or by comparing ratios of malar or flagella segment length [13], such morphometric features can be difficult to see in photos. Species-level identification usually requires careful examination under a microscope by experts. Consequently, reliably identified images of most bee species are rare or absent in crowdsourced datasets and are not available in sufficient numbers for training computer vision models. New sources of images for model training are needed.

Acquiring new annotated training images from the field is challenging because photographed individuals must also be captured and identified. However, existing museum or research collections, with pinned specimens that have been identified by experts are ideal for acquiring new images for computer vision model training. Pinned specimens can be quickly photographed under a microscope from standard angles under standard lighting conditions. This reduces the contextual variation of images taken in the field, which can confuse vision models and necessitate more images for effective model training [14].

In addition to images of whole specimens, images of bee wings alone may be effective for identifying bees [15–18]. Many bee species have distinctive patterns of venation on their wings that can be used in combination with other morphological features to help differentiate species [19]. For example, Hall [16] and Kozmus et al. [17] each mapped important nodes and cell centroids on bee forewings, and used k-nearest neighbor classification and discriminant analysis to effectively separate species. More recent deep learning techniques using convolutional neural networks (CNN) may be more flexible as they do not rely on predetermined feature input [20], such as the location of specific nodes. Like whole specimens, bee wing images may be acquired under standard conditions that minimize distracting background noise. Wing images are further standardized because they do not capture uninformative variation in the pose or condition of the rest of the body. With whole specimens, for example, bee hair can be variously matted, wings folded, and appendages positioned differently for each specimen. Photographing only wings in a flat plane reduces this type of variation, which could help improve the accuracy of identifications over images of whole bees. On the other hand, focusing on only a single part of the bee may provide fewer features for the model to differentiate among species.

We gathered new images of whole-bee specimens and bee wings for classification model training from our research collections. Our goals were to (1) assess the potential for species level identification of small and other challenging species through computer vision, and (2) to qualitatively compare the effectiveness of models trained on images of bee wings vs whole bee specimens. This was a qualitative comparison because the two image datasets were comprised largely of different species and sample sizes.

## Materials and methods

We imaged whole pinned bee specimens and bee wings from our research collections for computer vision model training.

### Pinned bee imaging

**Fig 1:**
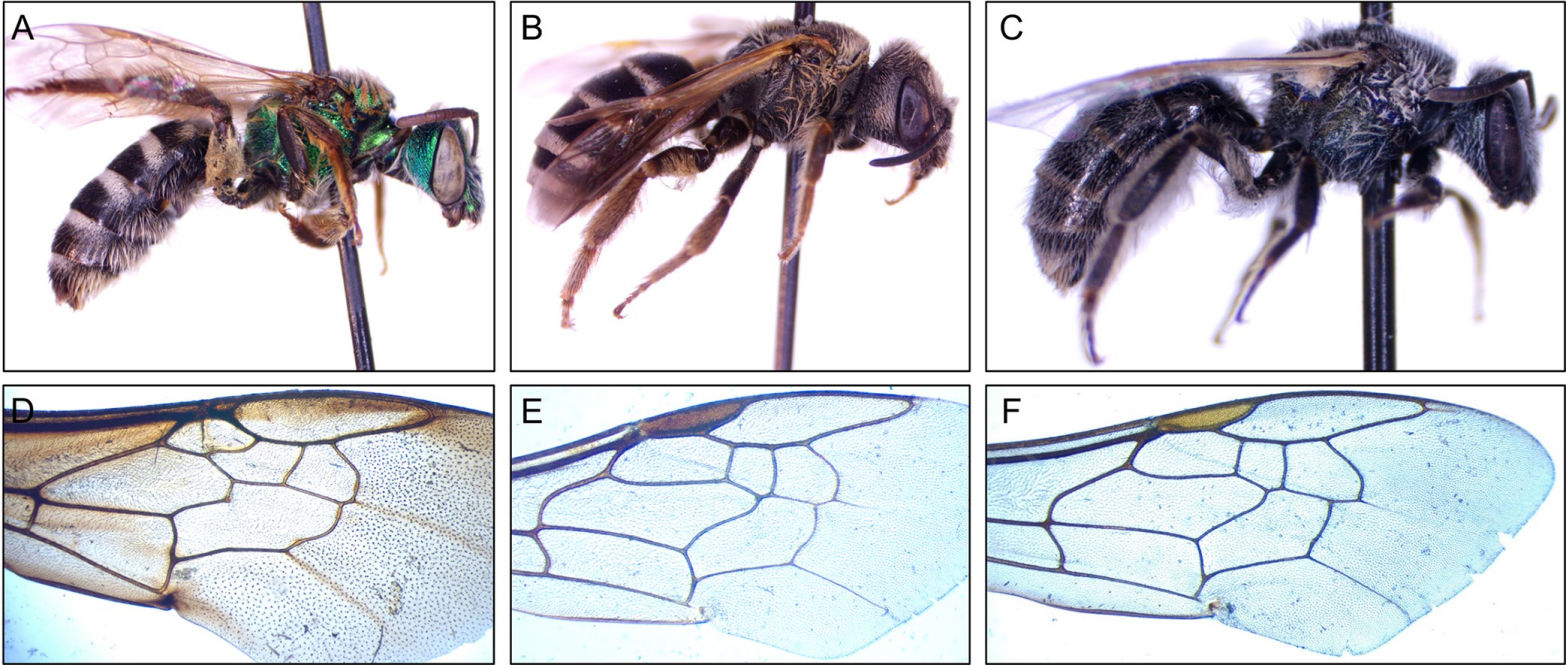
Example images of (A) *Agapostemon virescens*, (B) *Halictus ligatus*, (C) *Lasioglossum oceanicum*, (D) *Bombus griseocollis*, (E) *Lasioglossum leucozonium*, and (F) *Agapostemon texanus*. Images are not to relative scale and, except for cropping, are left unprocessed.

Pinned specimens were from ecological studies in Kansas [21,22] and Wisconsin USA (2), and were identified to species by taxonomic experts Michael Arduser and Jason Gibbs, respectively. We used a Nikon SMZ800N dissecting microscope (Nikon Metrology Inc.) with a 5-megapixel microscope camera (Swiftcam SC503, Swift Microscopes) mounted to a trinocular head. Specimens were imaged in front of a white background and illuminated by a 144-LED ring light (AmScope). We imaged females of 20 species from 6 genera from the families Halictidae and Apidae (Table 1). This set included one carpenter bee species (*Ceratina strenua*) and 19 sweat bee species commonly found in Midwestern grasslands, including 13 species of *Lasioglossum* (*Dialictus*) as well as *Lasioglossum* (*Hemihalictus*) *pectorale*. Example images for select species are shown in Fig 1 and examples for all species are shown S1 and S2 Figs in S1 File.

**Table 1:** Species list and mean species-level classification model performance for pinned bee images.

Species	Mean Precision	Mean Recall	Mean F1-score	Training images	Validation images	Test images	Total images
*Agapostemon virescens*	1.000	1.000	1.000	372	96	12	480
*Augochlorella aurata*	0.947	1.000	0.973	372	96	12	480
*Augochlorella persimilis*	1.000	0.944	0.971	372	96	12	480
*Augochloropsis metallica*	1.000	1.000	1.000	228	60	12	300
*Ceratina strenua*	1.000	1.000	1.000	372	96	12	480
*Halictus ligatus*	1.000	1.000	1.000	372	96	12	480
*Lasioglossum albipenne*	0.935	0.806	0.865	372	96	12	480
*Lasioglossum anomalum*	0.947	1.000	0.973	372	96	12	480
*Lasioglossum cressonii*	0.973	1.000	0.986	216	60	12	288
*Lasioglossum disparile*	0.900	1.000	0.947	372	96	12	480
*Lasioglossum hitchensi*	0.667	0.833	0.741	360	96	12	468
*Lasioglossum leucocomum*	1.000	0.639	0.780	372	96	12	480
*Lasioglossum oceanicum*	0.971	0.944	0.958	312	72	12	396
*Lasioglossum paradmirandum*	0.771	0.750	0.760	96	36	12	144
*Lasioglossum pectorale* ^1^	1.000	1.000	1.000	168	48	12	228
*Lasioglossum pilosum*	0.900	1.000	0.947	216	48	12	276
*Lasioglossum pruinosum*	0.972	0.861	0.913	372	96	12	480
*Lasioglossum semicaeruleum*	0.969	1.000	0.984	372	96	12	480
*Lasioglossum trigeminum*	0.973	1.000	0.986	372	96	12	480
*Lasioglossum versatum*	0.973	1.000	0.986	372	96	12	480

Precision, recall, and F1-scores are the mean of species-level scores averaged over the results from the three versions of the test dataset

^1^Subgenus *Leuchalictus*. All other *Lasioglossum* belong to the subgenus *Dialictus*.

We took 12 images per specimen: 6 images from the left and right-side parallel plane, left- and right-side oblique dorsal, left- and right-side oblique ventral. We repeated the process to obtain another 6 images from similar angles but looking down at ~20° angles. When available, we photographed 40 specimens per species. However, 6 of 20 species were represented by 12–33 specimens (Table 1). We limited images of pinned specimens to females as male specimens were not available in sufficient numbers to be included. This process resulted in a total of 8,340 images, which were divided into training (80%) and validation sets (20%). For each species, we randomly selected one individual from the training set and held out the 12 images belonging to that individual to form the test set. The training set was used to train the classification models, the validation set was used to evaluate the training process and adjust model parameters, and the test set, comprised of unseen images, was used to independently evaluate the final model. When dividing images, we ensured that no individuals shared images across training, validation, and test sets. Because our image dataset is relatively small, we constructed three versions for training and evaluation, each with different individuals making up the training, validation, and test sets. None of the test images were shared among the three versions. Consistency across the three versions would indicate that results were not likely dependent on the particular division of images in a set.

### Bee wing imaging

Images of bee forewings were gathered from the research collections of Claudio Gratton, Elena Gratton, and Heather Hines. Wings were from workers of 6 bumble bee species from the eastern United States that are part of the same broader mimicry complex (anterior half of the setae on the body yellow, posterior half black) and thus can be difficult to tell apart by color features. *Bombus bimaculatus*, *B*. *griseocollis*, and *B*. *impatiens* were from southern Wisconsin and were identified by Michael Arduser, Olivia Bernauer, and Jeremy Hemberger. Two *B*. *bimaculatus* wing images were from central Pennsylvania and were identified by Heather Hines. *Bombus vagans*, *B*. *sandersoni*, and *B*. *perplexus* (the *B*. *vagans* mimicry complex) workers were collected from central Pennsylvania. Most were identified using DNA barcodes, which are fully diagnostic, but identifications were further cross validated against malar space (aids distinction of *B*. *sandersoni* and *B*. *vagans*) and color traits (more distinctive in *B*. *perplexus*). Three *B*. *perplexus*, and four *B*. *sandersoni* individuals were identified based on morphology by Heather Hines. This is a submimicry group within the eastern U.S. that all have yellow setae across the thorax and the first two metasomal tergites with yellow setae, thus are unreliably diagnosed with color. DNA barcoding was performed using cytochrome oxidase I and identifications were confirmed against multiple reference sequences in GenBank, with procedural details and barcode data outlined in Gratton et al. [23]. Some of the wing images from *B*. *perplexus*, *B*. *sandersoni* and *B*. *vagans* were from male specimens (5 of 29, 19 of 92, and 1 of 38 images, respectively) and were distributed randomly among train, validation, and test sets. We also included female bees from the species *Agapostemon sericeus*, *A*. *texanus*, *Ceratina calcarata*, and 9 species in the genus *Lasioglossum*, all of which were identified by Michael Arduser. Images of most bee wings were acquired by placing whole bee specimens under a Leica stereo microscope fitted with a digital camera (www.jenoptik.us, Jenoptik ProgRes camera) and positioning wings between glass slides or over a sheet of white paper. For the *B*. *vagans* mimicry complex, a forewing was removed from each specimen and placed on an Epson Perfection v6000 photo scanner with an identifying label associated with the corresponding DNA barcoded bee.

In all, we imaged 18 species from 4 genera from the families Apidae and Halictidae. Nine *Lasioglossum* species were imaged, including 5 from the subgenus *Dialictus*, 2 from the subgenus *Lasioglossum*, and 2 from the subgenus *Leuchalictus*. Depending on specimen availability, we acquired 29–107 images per species (Table 2). We took a single image of both forewings from individuals except for those with highly damaged wings, but only took a single image from one wing for the *B*. *vagans* mimicry group. Our wing dataset included 1,069 total images, which were divided into training (80%) and validation (20%) sets, ensuring that wings from the same individual were not included in both sets. We then randomly selected 3 images from the training set to form the test set. As described for the pinned images, we constructed three separate versions of the dataset for training, validating, and testing. evaluation.

**Table 2:** Species list and mean species-level classification model performance for bee wing images.

Species	Mean Precision	Mean Recall	Mean F1-score	Training images	Validation images	Test images	Total images
*Agapostemon sericeus*	1.000	1.000	1.000	23	6	3	32
*Agapostemon texanus*	1.000	1.000	1.000	83	21	3	107
*Bombus bimaculatus*	0.917	1.000	0.952	37	10	3	50
*Bombus griseocollis*	1.000	0.889	0.933	31	9	3	43
*Bombus impatiens*	1.000	1.000	1.000	49	13	3	65
*Bombus perplexus*	1.000	1.000	1.000	20	6	3	29
*Bombus sandersoni*	1.000	1.000	1.000	70	19	3	92
*Bombus vagans*	1.000	1.000	1.000	28	7	3	38
*Ceratina calcarata*	1.000	1.000	1.000	60	16	3	79
*Lasioglossum acuminatum* ^2^	0.833	0.889	0.838	76	20	3	99
*Lasioglossum admirandum* ^1^	1.000	1.000	1.000	34	9	3	46
*Lasioglossum coriaceum* ^2^	0.917	0.889	0.886	35	9	3	47
*Lasioglossum leucozonium* ^3^	1.000	1.000	1.000	26	7	3	36
*Lasioglossum oceanicum* ^1^	1.000	1.000	1.000	31	8	3	42
*Lasioglossum pilosum* ^1^	1.000	1.000	1.000	64	17	3	84
*Lasioglossum versatum* ^1^	1.000	1.000	1.000	64	17	3	84
*Lasioglossum zephyrus* ^1^	1.000	1.000	1.000	45	12	3	60
*Lasioglossum zonulus* ^3^	1.000	0.889	0.933	26	7	3	36

Precision, recall, and F1-scores are the mean of species-level scores averaged over the results from the three versions of the test dataset

^1^Subgenus *Dialictus*

^2^Subgenus *Lasioglossum*

^3^Subgenus *Leuchalictus*.

### Image cropping

Bee wing images were manually cropped to the edge of the wing to reduce the number of uninformative background pixels. To speed up the cropping process for whole pinned specimens, we developed an object detection algorithm to locate the bee within the image and perform crops automatically. We used the YOLOv8l algorithm [24], trained on over 32,000 box-annotated images of pinned bee specimens, and achieved a mean average precision (mAP) score of 99.5%. Automatically cropped images were manually checked for quality before using as input for species classification model training.

### Classification model training

We trained convolutional neural networks (CNN), a class of artificial neural network, for species classification (reviewed by 20). A CNN is ideal for image classification because it does not rely on predefined features for making predictions, such as the morphological features used in a dichotomous key. Instead, a CNN "learns" its own feature set based only on a set of prelabeled images. Briefly, a CNN works by first passing an input image tensor through a series of filters to quantify feature maps at different pixel resolutions, i.e., the feature extraction or convolutional process. The feature information is then passed through a fully connected network, comprised of multiple layers of interconnected nodes (neurons) to arrive at a prediction. Equations, or activation functions based on connection weights and the ease of neuron firing, are used to determine the activation of network connections between nodes and thus how signals pass through the network. Prediction probabilities are determined by summing the strengths of connections to each class in the final layer of the network. In the training process, class predictions are compared to true class labels and a loss value, or error, is measured. Network parameters that contributed most to the loss value are then adjusted in a process called backpropagation, which is analogous to "learning". This proceeds iteratively each time a batch of data passes through the network and over a series of epochs (complete runs of the training dataset) with the goal of minimizing loss. For a general CNN overview, see Rawat and Wang [20] or Boroweic et al. [25] as it relates to ecology and evolution.

The classification models for both bee wing and whole specimen image sets were trained using the EfficientNetV2L [26] CNN architecture in a TensorFlow environment. EfficientNetV2 is a family of CNN that achieves state-of-the-art Top-1 accuracy on the ImageNet [27] dataset while maintaining a relatively small number of parameters and quick training time. As expected for EfficientNetV2L, we resized all our images to 480×480 pixels. Images were preprocessed by standardizing pixel values for each RGB band between 0 and 255. We used image augmentation to help reduce overfitting and increase the generality of the classification models: rotation ≤100°, height and width shift ≤ 0.2, shear range ≤ 0.2, zoom range ≤ 0.2, random horizontal flip, using nearest neighbors to backfill. Dropout, at a factor of 0.95, was used on the final dense layer to reduce overfitting. Classification probabilities were calculated using the softmax activation function. To help account for class imbalance, we weighted class predictions based on the number of training images per species. The model was compiled and optimized with stochastic gradient descent (SGD). Models were trained for 100 epochs with an initial learning rate of 0.01, which was reduced by a factor of 0.5 as the validation loss reached a plateau. We monitored validation loss after each epoch and, for each of the bee wing and pinned specimen training runs, selected the model with the lowest validation loss.

Using the test sets, we evaluated model performance based on overall accuracy, mean precision, recall, and F1 score. Overall accuracy of the test set is defined as the number of correct predictions divided by the total number of predictions. Precision indicates the likelihood that an unknown species is correctly predicted, and is calculated for each species as the number of true positives divided by the total number of positive predictions or True Positives/(True Positives + False Positives). We emphasize this metric because it is important from the perspective of someone trying to identify an unknown specimen. Recall (or sensitivity), on the other hand, indicates the likelihood that a known species will be correctly predicted, and is calculated for each species as True Positives/(True Positives + False Negatives). The F1-score blends precision and recall metrics and is calculated as (2 × precision × recall) / (precision + recall). We took the average of species-level scores to calculate mean precision, recall, and F1-score.

## Results

Accuracy and model loss values for the training and validation sets for both pinned specimens and bee wings saturated, at high and low points, respectively, near epoch 40 out of 100 across all training runs. There was no indication of overfitting. Because we trained the classification model on three different subsets of the pinned and wing datasets, we report mean performance scores. Performance scores for each subset are individually reported S1-S6 Tables in S1 File). Mean overall model accuracy of the test sets was 0.939 and 0.975 for pinned specimen and wing classifiers, respectively. The bee wing model performed somewhat better than the whole-bee model, even though its image dataset was only ~1/8 the size of the pinned bee dataset (Table 3).

**Table 3:** Classification model performance scores for each of the three test set versions of the pinned and wing datasets.

Dataset version	Overall model training accuracy	Overall model validation accuracy	Overall model test accuracy	Mean species-level test precision	Mean species-level test recall	Mean species-level test F1-score	Number training images	Number validation images	Number test images
Pinned 1	0.994	0.953	0.933	0.937	0.933	0.931	6432	1668	240
Pinned 2	0.996	0.953	0.946	0.953	0.946	0.943	6432	1668	240
Pinned 3	0.992	0.935	0.938	0.957	0.938	0.924	6432	1668	240
Mean	0.994	0.947	0.939	0.949	0.939	0.933	6432	1668	240
Wing 1	0.993	0.981	0.982	0.986	0.982	0.981	802	213	54
Wing 2	0.999	0.944	0.963	0.972	0.963	0.962	802	213	54
Wing 3	0.980	0.986	0.982	0.986	0.982	0.981	802	213	54
Mean	0.991	0.970	0.975	0.981	0.976	0.975	802	213	54

**Fig 2:**
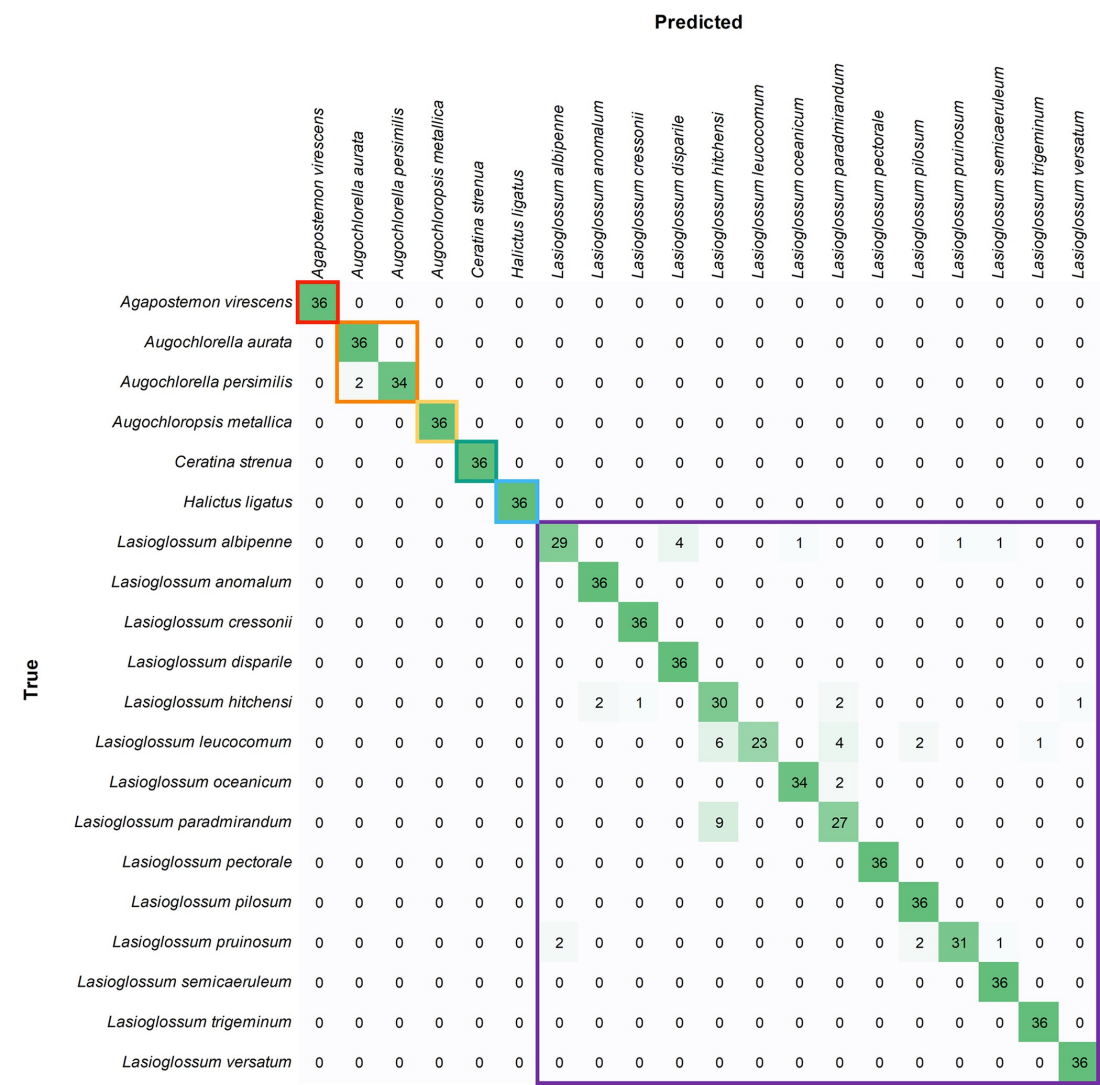
Pinned bee confusion matrix with results combined over the three test sets. Values represent the number of images for predicted and true (actual) species. Colored boxes outline the predictions within genera.

**Fig 3:**
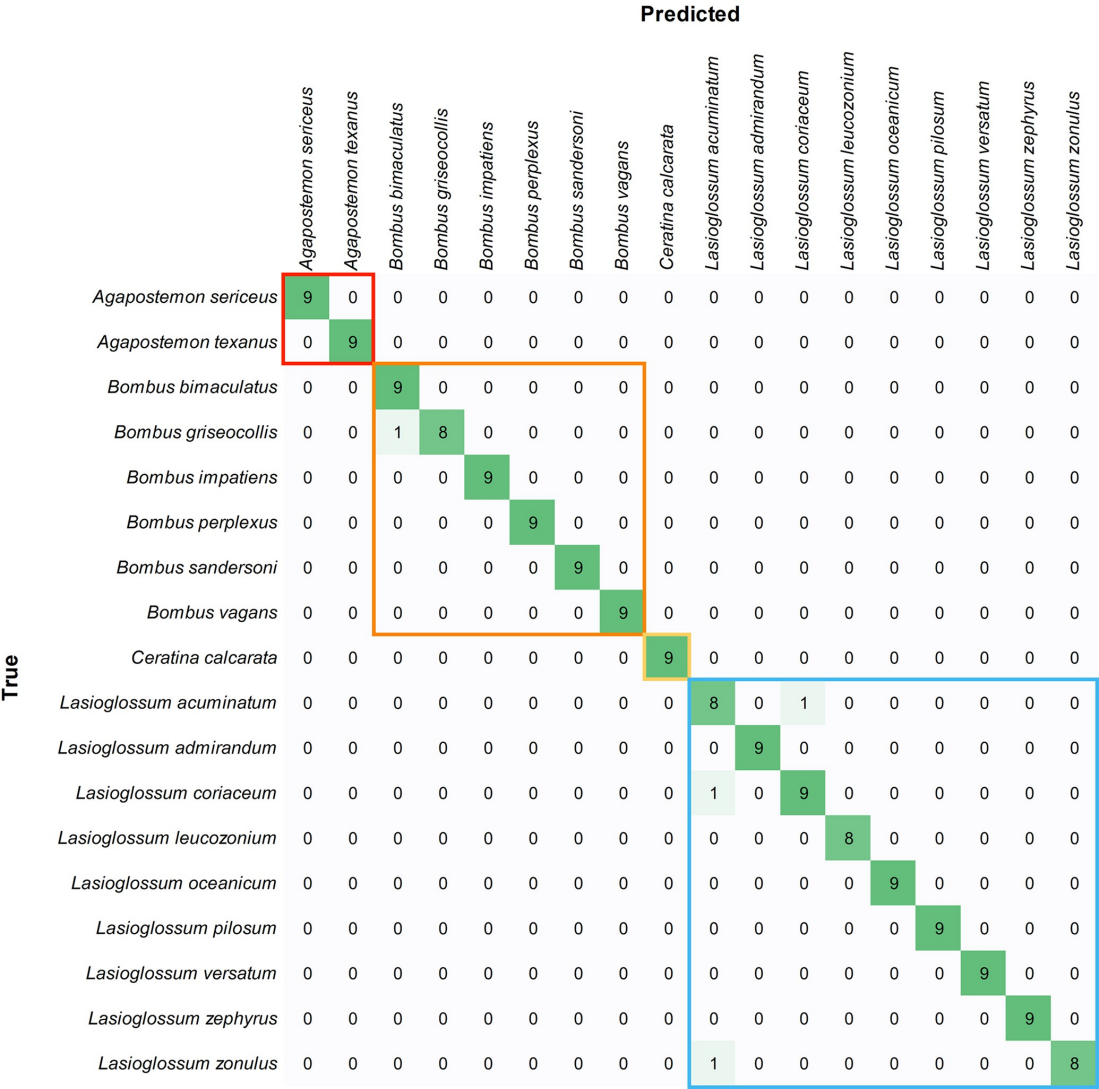
Bee wing confusion matrix with results combined over the three test sets. Values represent the number of images for predicted and true (actual) species. Colored boxes outline the predictions within genera.

Mean species-level precision was 0.949 and 0.981 for pinned and wing classifiers, respectively (Table 3). That is, given an unknown specimen, the correct species was predicted 94.9% and 98.1% of the time. Confusion matrices indicated that, for both datasets, prediction errors were limited to species within genera and that there were no prediction errors between genera (Figs 2 and 3; confusion matrices for each image subset are shown S3-S8 Figs in S1 File). Thus, the models were exceptional at separating genera in the test datasets.

The pinned bee classification model had outstanding performance for the 6 non-*Lasioglossum* species, with only two prediction errors across 216 test images (Table 1). Here, two images were predicted to be *Augochlorella aurata* that were actually *A*. *persimilis* (Fig 2). The model performed well on the 14 *Lasioglossum* species, with a mean precision of 0.925 on the test sets. Mean precision scores were relatively low for *L*. *hitchensi* (0.667) and *L*. *paradmirandum* (0.771). The remaining 12 *Lasioglossum* species had precision scores of 0.935 or greater.

On the other hand, *L*. *leucocomum* had high mean precision (1.000) but low mean recall (0.639). In other words, predictions of *L*. *leucocomum* were always correct, but predictions of *L*. *hitchensi* or *L*. *paradmirandum*, for example, were sometimes given as *L*. *leucocomum*. These three species accounted for much of the confusion between images of pinned specimens. *L*. *hitchensi* and *L*. *paradmirandum* may be more likely to be confused by humans than *L*. *leucocomum* as they belong to the same species group. Similarly, predictions of *L*. *disparile* were sometimes actually *L*. *albipenne*, and predictions of *L*. *albipenne* were sometimes actually *L*. *pruinosum*; three species that share a similar appearance [10].

The bee wing classification model performed well for *Lasioglossum* with a mean precision of 0.972 for this group (Table 2). One image that was predicted to be *L*. *acuminatum* was actually *L*. *coriaceum* and one image predicted to be *L*. *coriaceum* was actually *L*. *acuminatum*; two species that are from the same subgenus (*Lasioglossum*) and thus potentially more likely to be confused by an expert. On the other hand, one prediction of an *L*. acuminatum wing was actually *L*. *zonulus*, a member of a different subgenus (*Dialictus*), and therefore less likely to be confused by an expert looking at whole specimens. The remaining 6 *Lasioglossum* species, with a total of 54 test images, had no prediction errors.

*Bombus* species had a mean precision of 0.986 (Table 2). The one misclassified *Bombus* test image occurred between *B*. *bimaculatus* and *B*. *griseocollis*. There were no prediction errors among the 18 *Agapostemon* test images (Fig 3).

## Discussion

Recent studies have demonstrated the effectiveness of computer vision for studying pollinating insects [9,28], including identifying bee species in images [7,8]. This is especially true for large *Bombus* species for which there are sufficient training data available from community science programs like iNaturalist (https://www.inaturalist.org) and public repositories for such programs, like GBIF (https://www.gbif.org). With this study, we have shown that a deep learning approach is transferable to other taxa and can be applied to smaller bee species that are often more difficult to identify. Although we used two relatively small image datasets, we achieved high levels of mean precision for both pinned bee specimens (0.949) and bee wings (0.981). This is likely because images were relatively standardized in terms of lighting, background context, and pose, compared to the field-captured images that comprise most crowdsourced images used in CNN classification models. Our results suggest that image datasets could be quickly generated from museum and research collections for many of the thousands of species that are not well represented in public image repositories. Such training datasets would allow us to develop classification models for the rapid identification of curated bee specimens. However, future imaging efforts should include some of this type of variation in e.g., lighting and background so that end users of computer vision algorithms can use their own imaging equipment and not need to strictly conform to the imaging setup we employ here.

Qualitatively, pinned and wing classification models had similar performance. However, classification based on bee wings needed only one eighth the number of images to train an effective classifier. Focusing effort on imaging bee wings may allow training datasets to be generated more quickly. However, it can be challenging to efficiently photograph delicate wings from small bees without damaging them, and manipulation of such wings increases processing time. The wings of small specimens are also often damaged, crumpled, or folded over, reducing their utility as training images. Although, this may be less concerning for larger species with more robust wings, like *Bombus*. Images of whole specimens, on the other hand, are easier to acquire in the lab and, importantly, could be combined with field-based images for classification under different environmental contexts. Because bee wings in images from the field are often seen with motion blur or with venation obscured, combining lab-based bee wing images with field-based images of whole bees may not provide the same added benefit. That said, wing and whole-specimen image collection methods could each be effectively used for different research projects.

During the initial model evaluation process, one of our images labeled *B*. *perplexus* images was consistently classified as *B*. *sandersoni*. After examining the source data, we discovered that the image was mislabeled. Correcting the error and retraining the classification model slightly improved model performance. Spiesman et al. [7] discovered a similar error during the evaluation of a *Bombus* image classifier. This highlights potential alternative uses for our algorithms: independent error flagging and/or confirmation of human-made identifications.

**Fig 4:**
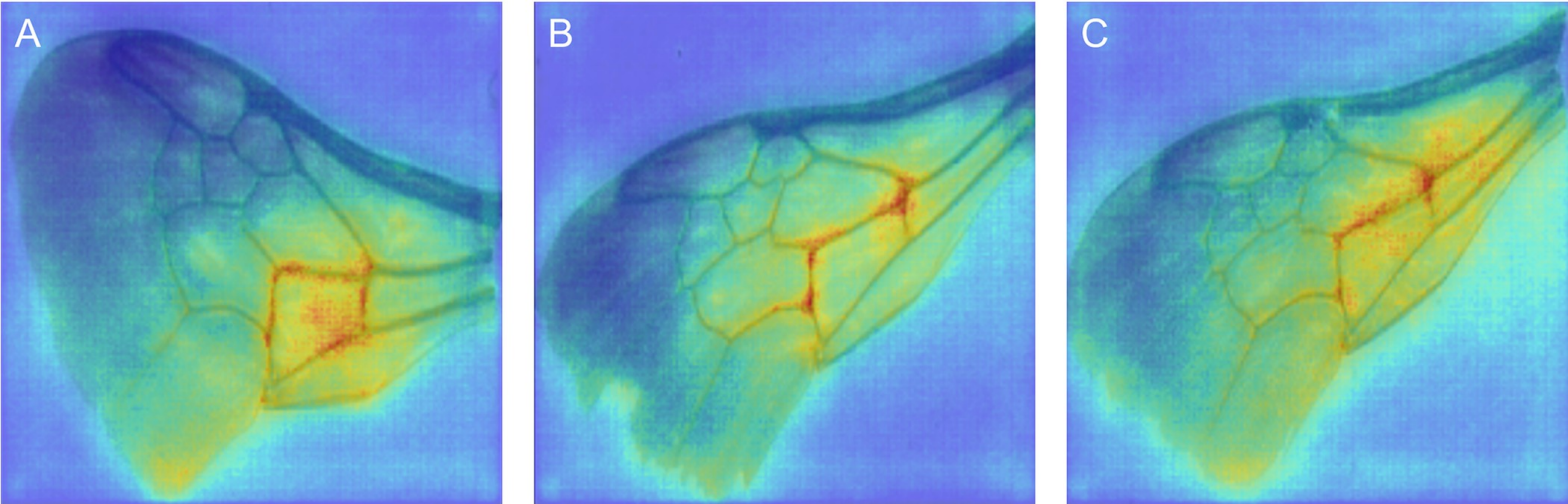
Example SmoothGrad images of wings from three different *Bombus sandersoni* specimens. Warmer colors indicate pixels that contributed more to the prediction. Red highlights around the second cubital cell suggest that this region is important for classifying these three images as *B*. *sandersoni*.

CNN classification models learn diagnostic features that are independent of taxonomic knowledge. For example, wing venation patterns, such as the number of submarginal cells or the degree of curvature of the basal vein, are used in taxonomic keys to help separate individuals among families and genera [19,29]. However, to make a species-level determinations, taxonomic keys often rely on multiple features from different parts of the specimen. Similarly, for whole specimens, taxonomists may rely on multiple images of multiple features that are not simultaneously captured in a single image. Yet our classifiers are generally able to make accurate predictions based on only a single image. Thus, it appears that CNN algorithm can learn important wing and body features for differentiating species that taxonomists do not utilize. Examining how vision algorithms focus their attention to make predictions may help us understand how these predictions are made and possibly help inform more effective ways to separate species. Attribution mapping, for example using Guided Backprop or SmoothGrad, can be used to highlight and analyze the fine-grained details in an image (i.e., which pixels) that are important for making a classification (Fig 4) [30,31].

Developing effective computer vision tools to identify difficult bee taxa, such as *Dialictus* and the *B*. *vagans* complex, will be important for generating better assessments of the abundance and distribution of species, recognizing which species are at risk, and understanding their diverse behaviors [10]. Barcode data from Pennsylvania bumble bees in the *B*. *vagans* mimicry group revealed these species to be historically frequently misidentified, as *B*. *sandersoni*, previously presumed to be rare, is actually the most abundant species [23]. This may have been amplified by misconceptions regarding the reliability of diagnostic color traits and assigning identity to the assumed more abundant taxon. In this case, wing scanning may be faster than morphometrics and cheaper than DNA barcoding for attaining bee identifications, especially for ecologists with less experience in subtle morphometric differences. Essentially, such scans may serve as time efficient and reliable morphological barcode (*sensu* 32).

Deep learning models have proven to be effective for many kinds of classification tasks [20,33–35], including bees and other insects [7,9,28,36]. The main factor limiting wider development and application of this technology appears to be the availability of labeled images for model training. Some of these images will come with expanded imaging efforts. However, the taxonomy of some bee taxa is not well understood. For example, we cannot develop reliable image datasets for genera such as *Nomada*, *Sphecodes*, and *Lasioglossum* of the western US, without first improving our species-level taxonomic knowledge of these groups [11,37]. Reliable diagnosis is necessary for effective training sets. The fundamental science of taxonomy is therefore essential to realizing the potential of computer vision for bee identification.

## Conclusions

This study is a promising step in advancing computer vision to help ease the challenge of bee species identification. Future work should expand the number of specimens and images per class for greater accuracy and generalizability, as well as the number of species represented, especially those that are difficult to discern, so that classification models can be used in basic and applied research. Future work should also explore whether some imaging angles provide more reliable predictions than others. Focusing on gathering the most informative images may increase the efficiency of generating image datasets and effectiveness of new classification models. For example, we did not include direct views of the face, which can be informative for bee species identification [e.g., 10,38]. Computer vision technology for taxonomic identification will continue to improve and become more reliable as new training data become available. However, our results suggest that the technology can already be used to enhance existing approaches for bee species identification.

## Supporting information

S1 File Supplementary figures and results. (DOCX)

## References

[1] Mallinger RE, Gratton C. Species richness of wild bees, but not the use of managed honeybees, increases fruit set of a pollinator-dependent crop. J Appl Ecol. 2015; 52(2):323–30. https://besjournals.onlinelibrary.wiley.com/doi/abs/10.1111/1365-2664.12377.

[2] Spiesman BJ, Bennett A, Isaacs R, Gratton C. Harvesting effects on wild bee communities in bioenergy grasslands depend on nesting guild. Ecol Appl. 2019; 29(2):e01828. https://esajournals.onlinelibrary.wiley.com/doi/abs/10.1002/eap.1828. 30412332 10.1002/eap.1828

[3] Graham KK, Gibbs J, Wilson J, May E, Isaacs R. Resampling of wild bees across fifteen years reveals variable species declines and recoveries after extreme weather. Agric Ecosyst Environ. 2021 Sep 1; 317:107470. https://www.sciencedirect.com/science/article/pii/S0167880921001742.

[4] Drew LW. Are we losing the science of taxonomy? As need grows, numbers and training are failing to keep up. BioScience. 2011 Dec 1; 61(12):942–6. https://academic.oup.com/bioscience/article/61/12/942/390232.

[5] Boyd RJ, Aizen MA, Barahona-Segovia RM, Flores-Prado L, Fontúrbel FE, Francoy TM, et al. Inferring trends in pollinator distributions across the Neotropics from publicly available data remains challenging despite mobilization efforts. Divers Distrib. 2022; 28(7):1404–15. https://onlinelibrary.wiley.com/doi/abs/10.1111/ddi.13551.

[6] Hemberger J, Crossley MS, Gratton C. Historical decrease in agricultural landscape diversity is associated with shifts in bumble bee species occurrence. Ecol Lett. 2021; 24(9):1800–13. http://onlinelibrary.wiley.com/doi/abs/10.1111/ele.13786. 34143928 10.1111/ele.13786

[7] Spiesman BJ, Gratton C, Hatfield RG, Hsu WH, Jepsen S, McCornack B, et al. Assessing the potential for deep learning and computer vision to identify bumble bee species from images. Sci Rep. 2021 Apr 7; 11(1):7580. https://www.nature.com/articles/s41598-021-87210-1. doi: 10.1038/s41598-021-87210-1 33828196 PMC8027374

[8] Suzuki-Ohno Y, Westfechtel T, Yokoyama J, Ohno K, Nakashizuka T, Kawata M, et al. Deep learning increases the availability of organism photographs taken by citizens in citizen science programs. Sci Rep. 2022 Jan 24; 12(1):1210. https://www.nature.com/articles/s41598-022-05163-5. doi: 10.1038/s41598-022-05163-5 35075168 PMC8786926

[9] Bjerge K, Alison J, Dyrmann M, Frigaard CE, Mann HMR, Høye TT. Accurate detection and identification of insects from camera trap images with deep learning. bioRxiv; 2022. p. 2022.10.25.513484. https://www.biorxiv.org/content/10.1101/2022.10.25.513484v1.

[10] Gibbs J. Revision of the metallic *Lasioglossum (Dialictus)* of eastern North America (Hymenoptera: Halictidae: Halictini). Zootaxa. 2011 Oct 28; 3073(1):1–216. https://www.biotaxa.org/Zootaxa/article/view/zootaxa.3073.1.1.

[11] Portman ZM, Bruninga-Socolar B, Cariveau DP. The state of bee monitoring in the United States: A call to refocus away from bowl traps and towards more effective methods. Ann Entomol Soc Am. 2020; 113(5): 337–342. https://academic.oup.com/aesa/advance-article/doi/10.1093/aesa/saaa010/5846123.

[12] Gibbs J. DNA barcoding a nightmare taxon: assessing barcode index numbers and barcode gaps for sweat bees. Genome. 2018; 61(1):21–31. https://cdnsciencepub.com/doi/10.1139/gen-2017-0096. 28972864 10.1139/gen-2017-0096

[13] Milam J, Johnson DE, Andersen JC, Fassler AB, Narango DL, Elkinton JS. Validating morphometrics with DNA barcoding to reliably separate three cryptic species of *Bombus* Cresson (Hymenoptera: Apidae). Insects. 2020; 11(10):669. https://www.mdpi.com/2075-4450/11/10/669. doi: 10.3390/insects11100669 33007903 PMC7600840

[14] Zhang J, Marszałek M, Lazebnik S, Schmid C. Local features and kernels for classification of texture and object categories: a comprehensive study. Int J Comput Vis. 2007 Jun 1; 73(2):213–38. 10.1007/s11263-006-9794-4.

[15] Steinhage V, Schröder S, Roth V, Cremers AB, Drescher W, Wittmann D. The Science of “Fingerprinting” Bees. Ger Res. 2006; 28(1):19–21. https://www.academia.edu/16346112/The_science_of_Fingerprinting_bees.

[16] Hall CJ. An Automated Approach to Bee Identification from Wing Venation. MSc thesis. University of Wisconsin—Madison; 2011. https://idbee.ece.wisc.edu/thesis_hall.pdf.

[17] Kozmus P, Virant-Doberlet M, Meglič V, Dovč P. Identification of *Bombus* species based on wing venation structure. Apidologie. 2011; 42(4):472–80. https://hal.archives-ouvertes.fr/hal-01003570.

[18] Gérard M, Martinet B, Dehon M, Rasmont P, Williams PH, Michez D. The utility of wing morphometrics for assigning type specimens to cryptic bumblebee species. Syst Entomol. 2020; 45(4):849–56. https://onlinelibrary.wiley.com/doi/abs/10.1111/syen.12430.

[19] Michener CD. The Bees of the World. 2nd ed. Baltimore, USA: Johns Hopkins University Press; 2007.

[20] Rawat W, Wang Z. Deep convolutional neural networks for image classification: a comprehensive review. Neural Comput. 2017 Sep 1; 29(9):2352–449. doi: 10.1162/NECO_a_00990 28599112

[21] Butters J, Murrell E, Spiesman BJ, Kim TN. Native flowering border crops attract high pollinator abundance and diversity, providing growers the opportunity to enhance pollination services. Environ Entomol. 2022 Apr 1; 51(2):492–504. doi: 10.1093/ee/nvac013 35298611

[22] Butters J. Providing for pollinators: conserving and integrating natural habitats to support pollinator conservation efforts. MSc thesis. Kansas State University; 2021. https://krex.k-state.edu/bitstream/handle/2097/41587/JessicaNicoleButters2021.pdf.

[23] Gratton EM, McNeil DJ Jr, Grozinger CM, Hines HM. Local habitat type influences bumble bee pathogen loads and bee species distributions. Environ Entomol. 2023 Jun 1; 52(3):491–501. doi: 10.1093/ee/nvad027 37133965

[24] Jocher G, Chaurasia A, Qiu J. Ultralytics YOLOv8. 2023. https://github.com/ultralytics/ultralytics.

[25] Borowiec ML, Dikow RB, Frandsen PB, McKeeken A, Valentini G, White AE. Deep learning as a tool for ecology and evolution. Methods Ecol Evol. 2022; 13(8):1640–60. https://onlinelibrary.wiley.com/doi/abs/10.1111/2041-210X.13901.

[26] Tan M, Le QV. EfficientNet: rethinking model scaling for convolutional neural networks. arXiv:190511946. 2019 Nov 22; http://arxiv.org/abs/1905.11946

[27] Deng J, Dong W, Socher R, Li LJ, Kai Li, Fei-Fei Li. ImageNet: A large-scale hierarchical image database. In: 2009 IEEE Conference on Computer Vision and Pattern Recognition. 2009. p. 248–55. https://ieeexplore.ieee.org/document/5206848.

[28] Høye TT, Ärje J, Bjerge K, Hansen OLP, Iosifidis A, Leese F, et al. Deep learning and computer vision will transform entomology. Proc Natl Acad Sci. 2021 Jan 12; 118(2). https://www.pnas.org/content/118/2/e2002545117. doi: 10.1073/pnas.2002545117 33431561 PMC7812775

[29] Gibbs J. Atypical wing venation in *Dialictus* and *Hemihalictus* and its implications for subgeneric classification of *Lasioglossum*. Psyche J Entomol. 2010 Oct 26; 2010:e605390. https://www.hindawi.com/journals/psyche/2010/605390/.

[30] Springenberg JT, Dosovitskiy A, Brox T, Riedmiller M. Striving for simplicity: the all convolutional net. arXiv:1412.6806; 2015. http://arxiv.org/abs/1412.6806.

[31] Smilkov D, Thorat N, Kim B, Viégas F, Wattenberg M. SmoothGrad: removing noise by adding noise. arXiv; 1706.03825vl. 2017. http://arxiv.org/abs/1706.03825.

[32] Ulmer JM, Mikó I, Deans AR, Krogmann L. The Waterston’s evaporatorium of Ceraphronidae (Ceraphronoidea, Hymenoptera): A morphological barcode to a cryptic taxon. J Hymenopt Res. 2021 Aug 31; 85:29–56. https://jhr.pensoft.net/article/67165/.

[33] Brinker TJ, Hekler A, Utikal JS, Grabe N, Schadendorf D, Klode J, et al. Skin cancer classification using convolutional neural networks: systematic review. J Med Internet Res. 2018 Oct 17; 20(10):e11936. https://www.jmir.org/2018/10/e11936. doi: 10.2196/11936 30333097 PMC6231861

[34] Kattenborn T, Leitloff J, Schiefer F, Hinz S. Review on convolutional neural networks (CNN) in vegetation remote sensing. ISPRS J Photogramm Remote Sens. 2021 Mar 1; 173:24–49. https://www.sciencedirect.com/science/article/pii/S0924271620303488.

[35] Gupta A, Anpalagan A, Guan L, Khwaja AS. Deep learning for object detection and scene perception in self-driving cars: Survey, challenges, and open issues. Array. 2021 Jul 1; 10:100057. https://www.sciencedirect.com/science/article/pii/S2590005621000059.

[36] Grijalva I, Spiesman BJ, McCornack B. Image classification of sugarcane aphid density using deep convolutional neural networks. Smart Agric Technol. 2023 Feb 1; 3:100089. https://www.sciencedirect.com/science/article/pii/S2772375522000545.

[37] Gibbs J, Ascher JS, Rightmyer MG, Isaacs R. The bees of Michigan (Hymenoptera: Apoidea: Anthophila), with notes on distribution, taxonomy, pollination, and natural history. Zootaxa. 2017 Nov 21; 4352(1):1–160. https://www.biotaxa.org/Zootaxa/article/view/zootaxa.4352.1.1. doi: 10.11646/zootaxa.4352.1.1 29245534

[38] Rightmyer M, Griswold T, Arduser M. A review of the non-metallic *Osmia* (*Melanosmia*) found in North America, with additional notes on palearctic *Melanosmia* (Hymenoptera, Megachilidae). ZooKeys. 2010 Jul 10; 60:37–77. https://zookeys.pensoft.net/article/2362. doi: 10.3897/zookeys.60.484 21594200 PMC3088345

