# Supplementary material for: Deep learning for identifying bee species from images of wings and pinned specimens
Source: PLoS One. 2024 May 28;19(5):e0303383. doi: 10.1371/journal.pone.0303383 (PMC11132477; doi:10.1371/journal.pone.0303383)
Supplement: S1 File — (DOCX) [file pone.0303383.s001.docx]

**Supplementary Tables and Figures**

**S1 Table. Classification model performance metrics for pinned bee images on test set 1.**

| **Species** | **Precision** | **Recall** | **F1-score** | **Number test images** |
| --- | --- | --- | --- | --- |
| *Agapostemon virescens* | 1.000 | 1.000 | 1.000 | 12 |
| *Augochlorella aurata* | 0.857 | 1.000 | 0.923 | 12 |
| *Augochlorella persimilis* | 1.000 | 0.833 | 0.909 | 12 |
| *Augochloropsis metallica* | 1.000 | 1.000 | 1.000 | 12 |
| *Ceratina strenua* | 1.000 | 1.000 | 1.000 | 12 |
| *Halictus ligatus* | 1.000 | 1.000 | 1.000 | 12 |
| *Lasioglossum albipenne* | 0.846 | 0.917 | 0.880 | 12 |
| *Lasioglossum anomalum* | 0.857 | 1.000 | 0.923 | 12 |
| *Lasioglossum cressonii* | 1.000 | 1.000 | 1.000 | 12 |
| *Lasioglossum disparile* | 1.000 | 1.000 | 1.000 | 12 |
| *Lasioglossum hitchensi* | 0.615 | 0.667 | 0.640 | 12 |
| *Lasioglossum leucocomum* | 1.000 | 1.000 | 1.000 | 12 |
| *Lasioglossum oceanicum* | 0.923 | 1.000 | 0.960 | 12 |
| *Lasioglossum paradmirandum* | 0.778 | 0.583 | 0.667 | 12 |
| *Lasioglossum pectorale* | 1.000 | 1.000 | 1.000 | 12 |
| *Lasioglossum pilosum* | 0.857 | 1.000 | 0.923 | 12 |
| *Lasioglossum pruinosum* | 1.000 | 0.667 | 0.800 | 12 |
| *Lasioglossum semicaeruleum* | 1.000 | 1.000 | 1.000 | 12 |
| *Lasioglossum trigeminum* | 1.000 | 1.000 | 1.000 | 12 |
| *Lasioglossum versatum* | 1.000 | 1.000 | 1.000 | 12 |
|  |  |  |  |  |
| Mean/Total | 0.937 | 0.933 | 0.931 | 240 |
| Overall test accuracy | 0.933 |  |  |  |

**S2 Table. Classification model performance metrics for pinned bee images on test set 2.**

| **Species** | **Precision** | **Recall** | **F1-score** | **Number test images** |
| --- | --- | --- | --- | --- |
| *Agapostemon virescens* | 1.000 | 1.000 | 1.000 | 12 |
| *Augochlorella aurata* | 1.000 | 1.000 | 1.000 | 12 |
| *Augochlorella persimilis* | 1.000 | 1.000 | 1.000 | 12 |
| *Augochloropsis metallica* | 1.000 | 1.000 | 1.000 | 12 |
| *Ceratina strenua* | 1.000 | 1.000 | 1.000 | 12 |
| *Halictus ligatus* | 1.000 | 1.000 | 1.000 | 12 |
| *Lasioglossum albipenne* | 1.000 | 0.500 | 0.667 | 12 |
| *Lasioglossum anomalum* | 1.000 | 1.000 | 1.000 | 12 |
| *Lasioglossum cressonii* | 0.923 | 1.000 | 0.960 | 12 |
| *Lasioglossum disparile* | 0.750 | 1.000 | 0.857 | 12 |
| *Lasioglossum hitchensi* | 0.833 | 0.833 | 0.833 | 12 |
| *Lasioglossum leucocomum* | 1.000 | 0.833 | 0.909 | 12 |
| *Lasioglossum oceanicum* | 1.000 | 1.000 | 1.000 | 12 |
| *Lasioglossum paradmirandum* | 1.000 | 0.833 | 0.909 | 12 |
| *Lasioglossum pectorale* | 1.000 | 1.000 | 1.000 | 12 |
| *Lasioglossum pilosum* | 0.857 | 1.000 | 0.923 | 12 |
| *Lasioglossum pruinosum* | 0.917 | 0.917 | 0.917 | 12 |
| *Lasioglossum semicaeruleum* | 0.857 | 1.000 | 0.923 | 12 |
| *Lasioglossum trigeminum* | 1.000 | 1.000 | 1.000 | 12 |
| *Lasioglossum versatum* | 0.923 | 1.000 | 0.960 | 12 |
|  |  |  |  |  |
| Mean/Total | 0.953 | 0.946 | 0.943 | 240 |
| Overall test accuracy | 0.946 |  |  |  |

**S3 Table. Classification model performance metrics for pinned bee images on test set 3.**

| **Species** | **Precision** | **Recall** | **F1-score** | **Number test images** |
| --- | --- | --- | --- | --- |
| *Agapostemon virescens* | 1.000 | 1.000 | 1.000 | 12 |
| *Augochlorella aurata* | 1.000 | 1.000 | 1.000 | 12 |
| *Augochlorella persimilis* | 1.000 | 1.000 | 1.000 | 12 |
| *Augochloropsis metallica* | 1.000 | 1.000 | 1.000 | 12 |
| *Ceratina strenua* | 1.000 | 1.000 | 1.000 | 12 |
| *Halictus ligatus* | 1.000 | 1.000 | 1.000 | 12 |
| *Lasioglossum albipenne* | 1.000 | 1.000 | 1.000 | 12 |
| *Lasioglossum anomalum* | 1.000 | 1.000 | 1.000 | 12 |
| *Lasioglossum cressonii* | 1.000 | 1.000 | 1.000 | 12 |
| *Lasioglossum disparile* | 1.000 | 1.000 | 1.000 | 12 |
| *Lasioglossum hitchensi* | 0.600 | 1.000 | 0.750 | 12 |
| *Lasioglossum leucocomum* | 1.000 | 0.083 | 0.154 | 12 |
| *Lasioglossum oceanicum* | 1.000 | 0.833 | 0.909 | 12 |
| *Lasioglossum paradmirandum* | 0.625 | 0.833 | 0.714 | 12 |
| *Lasioglossum pectorale* | 1.000 | 1.000 | 1.000 | 12 |
| *Lasioglossum pilosum* | 1.000 | 1.000 | 1.000 | 12 |
| *Lasioglossum pruinosum* | 1.000 | 1.000 | 1.000 | 12 |
| *Lasioglossum semicaeruleum* | 1.000 | 1.000 | 1.000 | 12 |
| *Lasioglossum trigeminum* | 0.923 | 1.000 | 0.960 | 12 |
| *Lasioglossum versatum* | 1.000 | 1.000 | 1.000 | 12 |
|  |  |  |  |  |
| Mean/Total | 0.957 | 0.938 | 0.924 | 240 |
| Overall test accuracy | 0.938 |  |  |  |

**S4 Table. Classification model performance metrics for bee wing images on test set 1.**

| **Species** | **Precision** | **Recall** | **F1-score** | **Number test images** |
| --- | --- | --- | --- | --- |
| *Agapostemon sericeus* | 1.000 | 1.000 | 1.000 | 3 |
| *Agapostemon texanus* | 1.000 | 1.000 | 1.000 | 3 |
| *Bombus bimaculatus* | 1.000 | 1.000 | 1.000 | 3 |
| *Bombus griseocollis* | 1.000 | 1.000 | 1.000 | 3 |
| *Bombus impatiens* | 1.000 | 1.000 | 1.000 | 3 |
| *Bombus perplexus* | 1.000 | 1.000 | 1.000 | 3 |
| *Bombus sandersoni* | 1.000 | 1.000 | 1.000 | 3 |
| *Bombus vagans* | 1.000 | 1.000 | 1.000 | 3 |
| *Ceratina calcarata* | 1.000 | 1.000 | 1.000 | 3 |
| *Lasioglossum acuminatum* | 1.000 | 0.667 | 0.800 | 3 |
| *Lasioglossum admirandum* | 1.000 | 1.000 | 1.000 | 3 |
| *Lasioglossum coriaceum* | 0.750 | 1.000 | 0.857 | 3 |
| *Lasioglossum leucozonium* | 1.000 | 1.000 | 1.000 | 3 |
| *Lasioglossum oceanicum* | 1.000 | 1.000 | 1.000 | 3 |
| *Lasioglossum pilosum* | 1.000 | 1.000 | 1.000 | 3 |
| *Lasioglossum versatum* | 1.000 | 1.000 | 1.000 | 3 |
| *Lasioglossum zephyrus* | 1.000 | 1.000 | 1.000 | 3 |
| *Lasioglossum zonulus* | 1.000 | 1.000 | 1.000 | 3 |
|  |  |  |  |  |
| Mean/Total | 0.986 | 0.982 | 0.981 | 54 |
| Overall test accuracy | 0.982 |  |  |  |

**S5 Table. Classification model performance metrics for bee wing images on test set 2.**

| **Species** | **Precision** | **Recall** | **F1-score** | **Number test images** |
| --- | --- | --- | --- | --- |
| *Agapostemon sericeus* | 1.000 | 1.000 | 1.000 | 3 |
| *Agapostemon texanus* | 1.000 | 1.000 | 1.000 | 3 |
| *Bombus bimaculatus* | 0.750 | 1.000 | 0.857 | 3 |
| *Bombus griseocollis* | 1.000 | 0.667 | 0.800 | 3 |
| *Bombus impatiens* | 1.000 | 1.000 | 1.000 | 3 |
| *Bombus perplexus* | 1.000 | 1.000 | 1.000 | 3 |
| *Bombus sandersoni* | 1.000 | 1.000 | 1.000 | 3 |
| *Bombus vagans* | 1.000 | 1.000 | 1.000 | 3 |
| *Ceratina calcarata* | 1.000 | 1.000 | 1.000 | 3 |
| *Lasioglossum acuminatum* | 0.750 | 1.000 | 0.857 | 3 |
| *Lasioglossum admirandum* | 1.000 | 1.000 | 1.000 | 3 |
| *Lasioglossum coriaceum* | 1.000 | 1.000 | 1.000 | 3 |
| *Lasioglossum leucozonium* | 1.000 | 1.000 | 1.000 | 3 |
| *Lasioglossum oceanicum* | 1.000 | 1.000 | 1.000 | 3 |
| *Lasioglossum pilosum* | 1.000 | 1.000 | 1.000 | 3 |
| *Lasioglossum versatum* | 1.000 | 1.000 | 1.000 | 3 |
| *Lasioglossum zephyrus* | 1.000 | 1.000 | 1.000 | 3 |
| *Lasioglossum zonulus* | 1.000 | 0.667 | 0.800 | 3 |
|  |  |  |  |  |
| Mean/Total | 0.972 | 0.963 | 0.962 | 54 |
| Overall test accuracy | 0.963 |  |  |  |

**S6 Table. Classification model performance metrics for bee wing images on test set 3.**

| **Species** | **Precision** | **Recall** | **F1-score** | **Number test images** |
| --- | --- | --- | --- | --- |
| *Agapostemon sericeus* | 1.000 | 1.000 | 1.000 | 3 |
| *Agapostemon texanus* | 1.000 | 1.000 | 1.000 | 3 |
| *Bombus bimaculatus* | 1.000 | 1.000 | 1.000 | 3 |
| *Bombus griseocollis* | 1.000 | 1.000 | 1.000 | 3 |
| *Bombus impatiens* | 1.000 | 1.000 | 1.000 | 3 |
| *Bombus perplexus* | 1.000 | 1.000 | 1.000 | 3 |
| *Bombus sandersoni* | 1.000 | 1.000 | 1.000 | 3 |
| *Bombus vagans* | 1.000 | 1.000 | 1.000 | 3 |
| *Ceratina calcarata* | 1.000 | 1.000 | 1.000 | 3 |
| *Lasioglossum acuminatum* | 0.750 | 1.000 | 0.857 | 3 |
| *Lasioglossum admirandum* | 1.000 | 1.000 | 1.000 | 3 |
| *Lasioglossum coriaceum* | 1.000 | 0.667 | 0.800 | 3 |
| *Lasioglossum leucozonium* | 1.000 | 1.000 | 1.000 | 3 |
| *Lasioglossum oceanicum* | 1.000 | 1.000 | 1.000 | 3 |
| *Lasioglossum pilosum* | 1.000 | 1.000 | 1.000 | 3 |
| *Lasioglossum versatum* | 1.000 | 1.000 | 1.000 | 3 |
| *Lasioglossum zephyrus* | 1.000 | 1.000 | 1.000 | 3 |
| *Lasioglossum zonulus* | 1.000 | 1.000 | 1.000 | 3 |
|  |  |  |  |  |
| Mean/Total | 0.986 | 0.982 | 0.981 | 54 |
| Overall test accuracy | 0.982 |  |  |  |


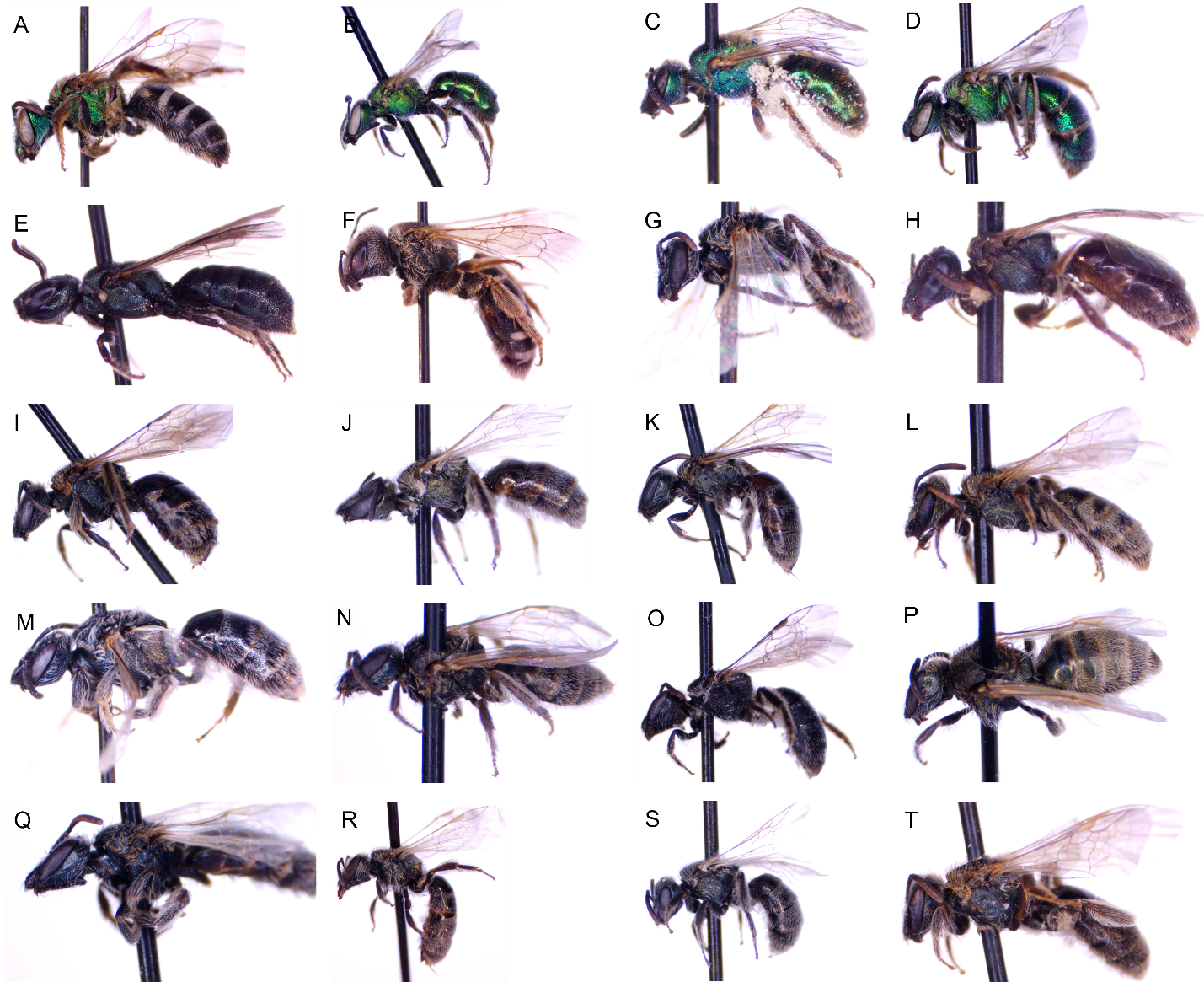


**S1 Fig. Example pinned specimen images for each species.** (A) *Agapostemon virescens*, (B) *Augochlorella aurata*, (C) *Augochlorella persimilis*, (D) *Augochloropsis metallica*, (E) *Ceratina strenua*, (F) *Halictus ligatus*, (G) *Lasioglossum albipenne*, (H) *Lasioglossum anomalum*, (I) *Lasioglossum cressonii*, (J) *Lasioglossum disparile*, (K) *Lasioglossum hitchensi*, (L) *Lasioglossum leucocomum*, (M) *Lasioglossum oceanicum*, (N) *Lasioglossum paradmirandum*, (O) *Lasioglossum pectorale*, (P) *Lasioglossum pilosum*, (Q) *Lasioglossum pruinosum*, (R) *Lasioglossum semicaeruleum*, (S) *Lasioglossum trigeminum*, and (T) *Lasioglossum versatum*. Specimens are not to relative scale and images have not been preprocessed.

**
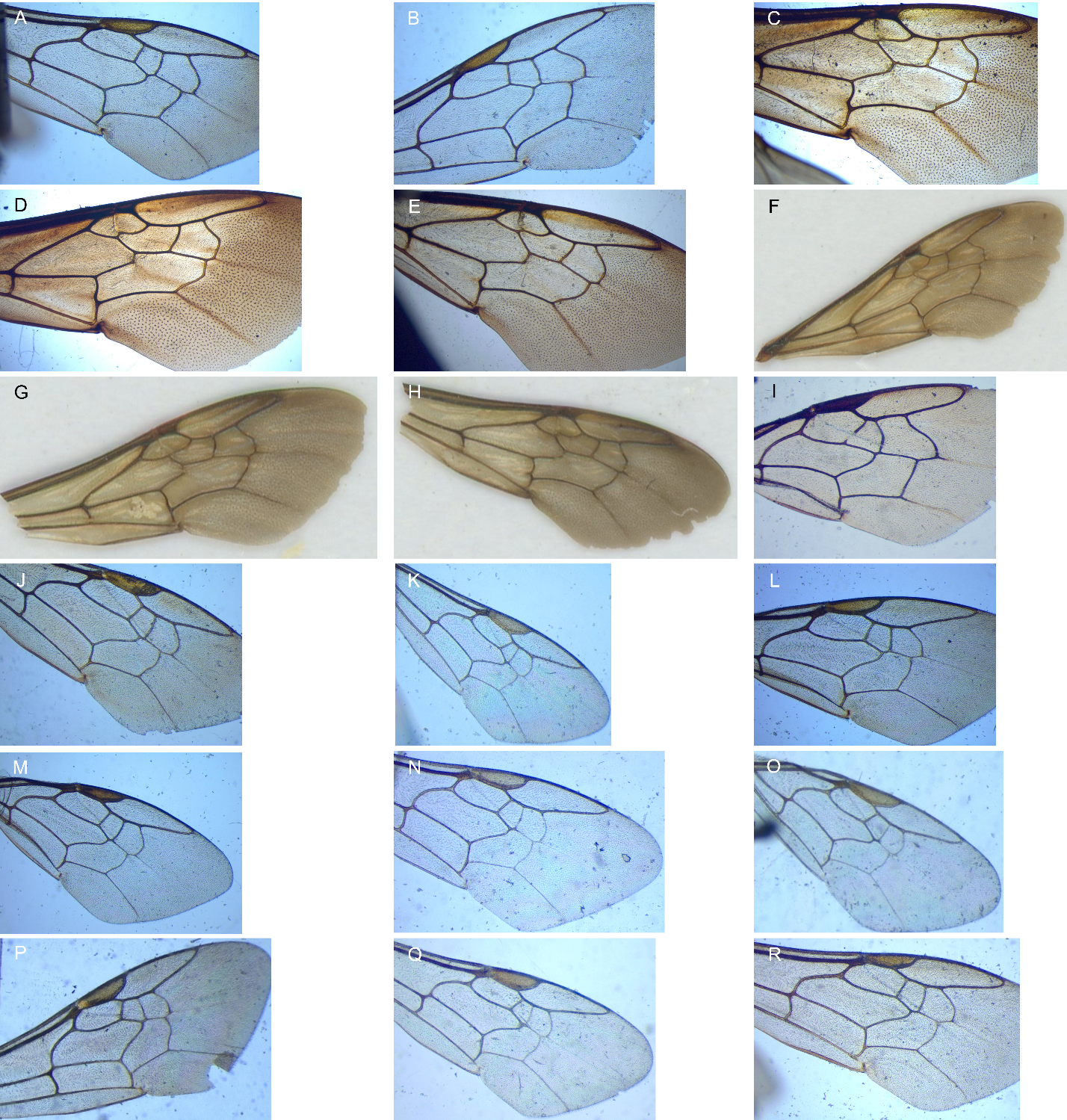
**

**S2 Fig. Example bee wing images for each species.** (A) *Agapostemon sericeus*, (B) *Agapostemon texanus*, (C) *Bombus bimaculatus*, (D) *Bombus griseocollis*, (E) *Bombus impatiens*, (F) *Bombus perplexus*, (G) *Bombus sandersoni*, (H) *Bombus vagans*, (I) *Ceratina calcarata*, (J) *Lasioglossum acuminatum*, (K) *Lasioglossum* *admirandum*, (L) *Lasioglossum coriaceum*, (M) *Lasioglossum* *leucozonium*, (N) *Lasioglossum oceanicum*, (O) *Lasioglossum pilosum*, (P) *Lasioglossum versatum*, (Q) *Lasioglossum zephyrus*, and (R) *Lasioglossum zonulus*. Wings are not to relative scale and images have not been preprocessed.


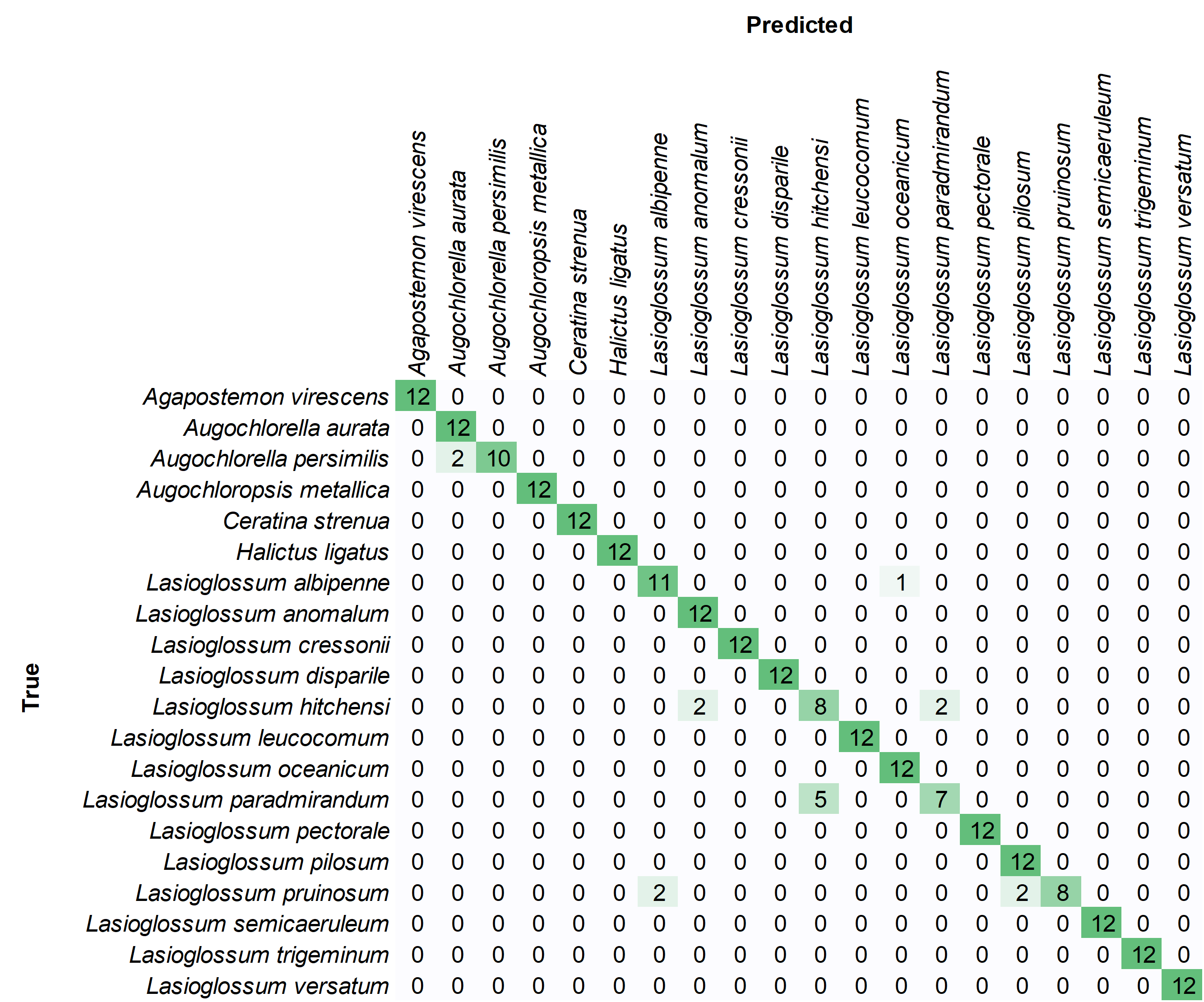


**S3 Fig. Confusion matrix for pinned specimen image results, test set 1.**


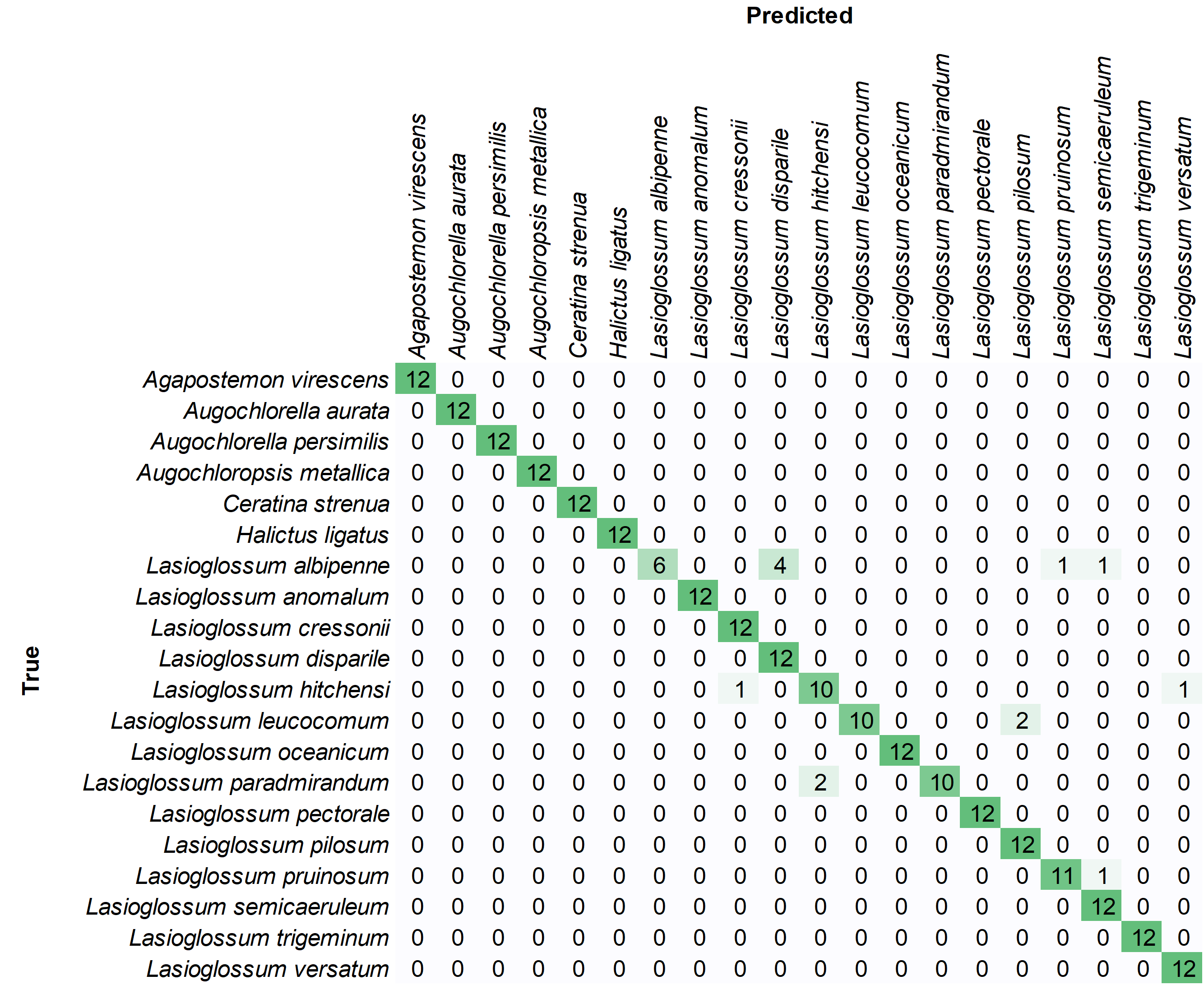


**S4 Fig. Confusion matrix for pinned specimen image results, test set 2.**


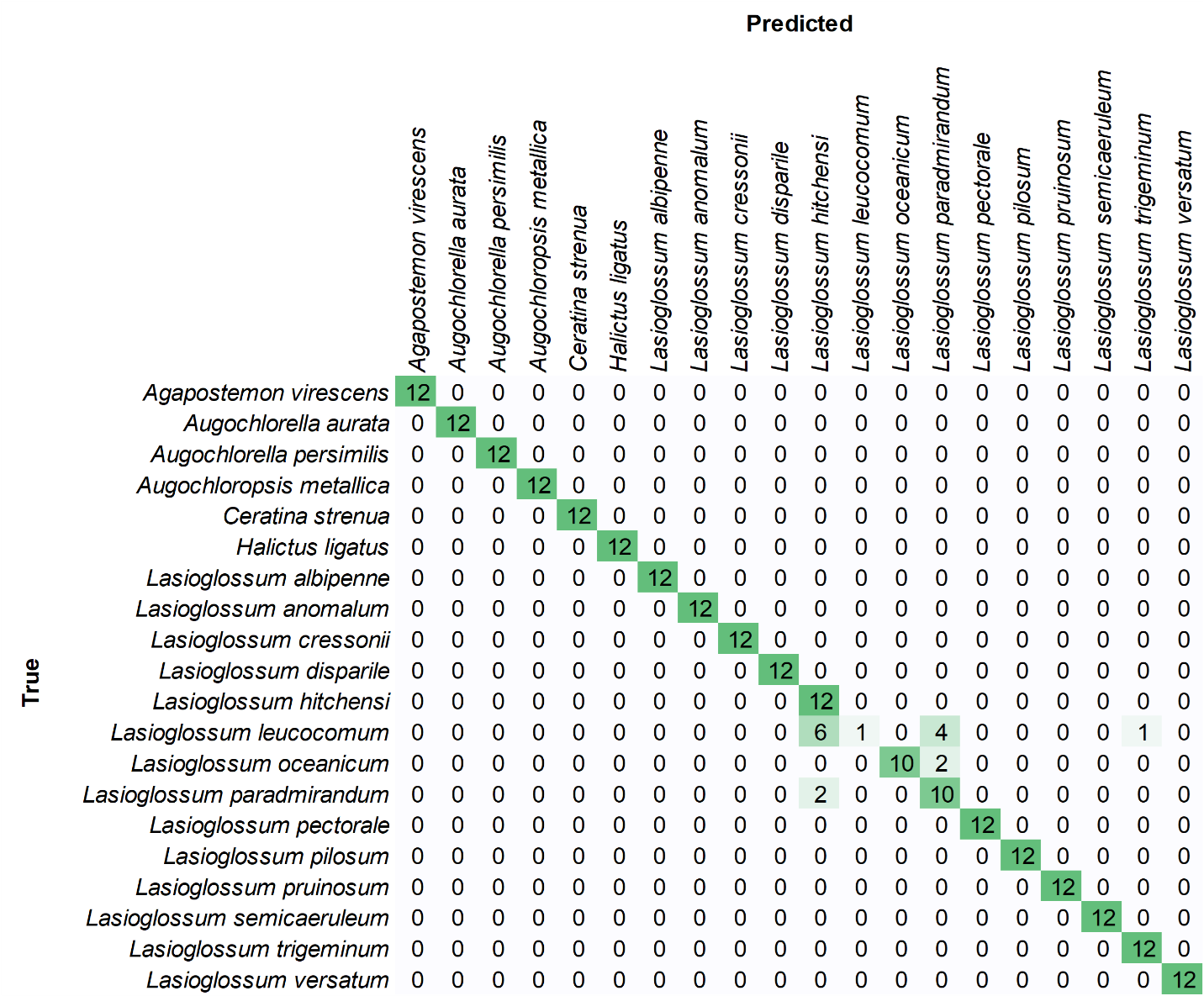


**S5 Fig. Confusion matrix for pinned specimen image results, test set 3.**


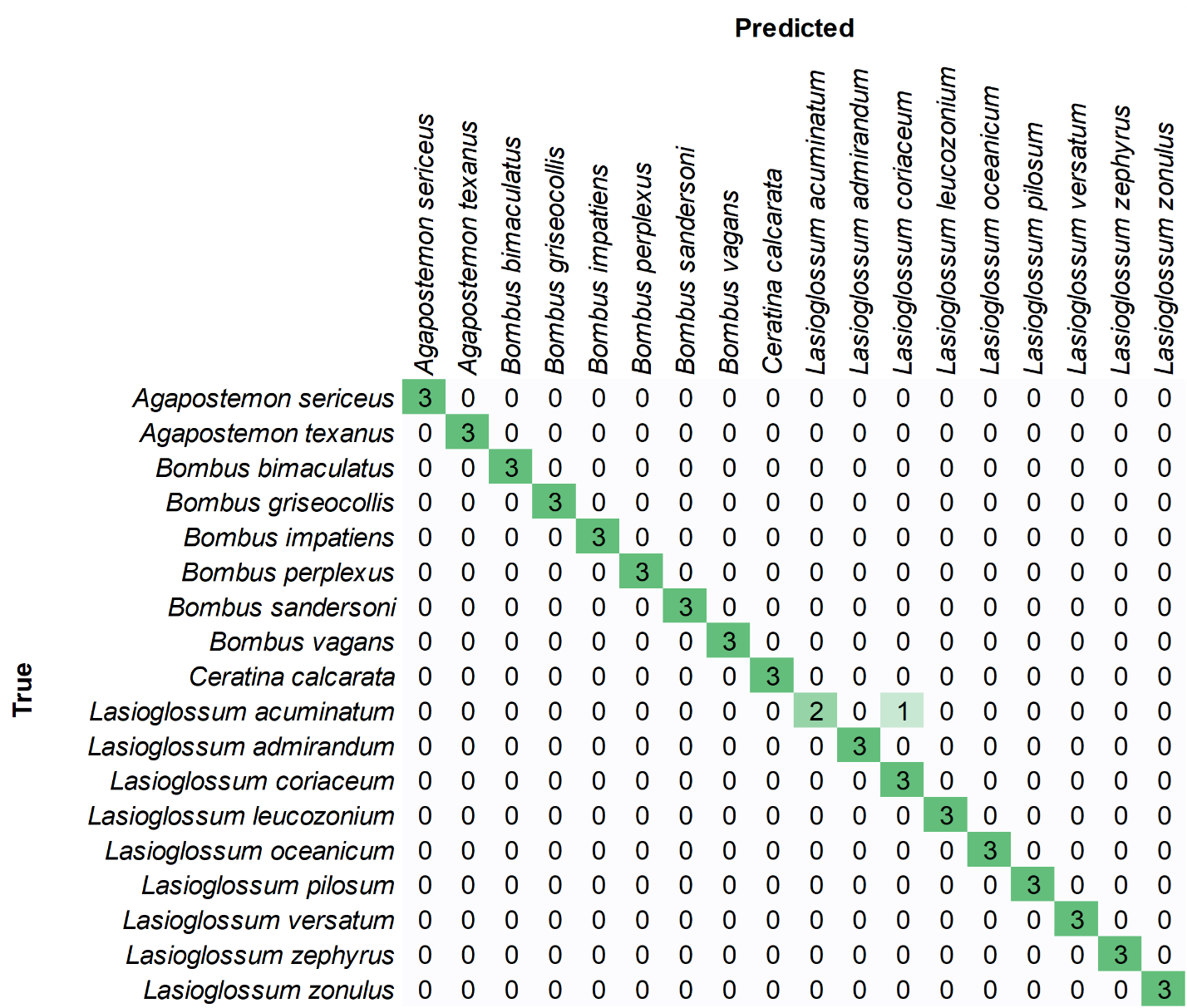


**S6 Fig. Confusion matrix for bee wing image results, test set 1.**


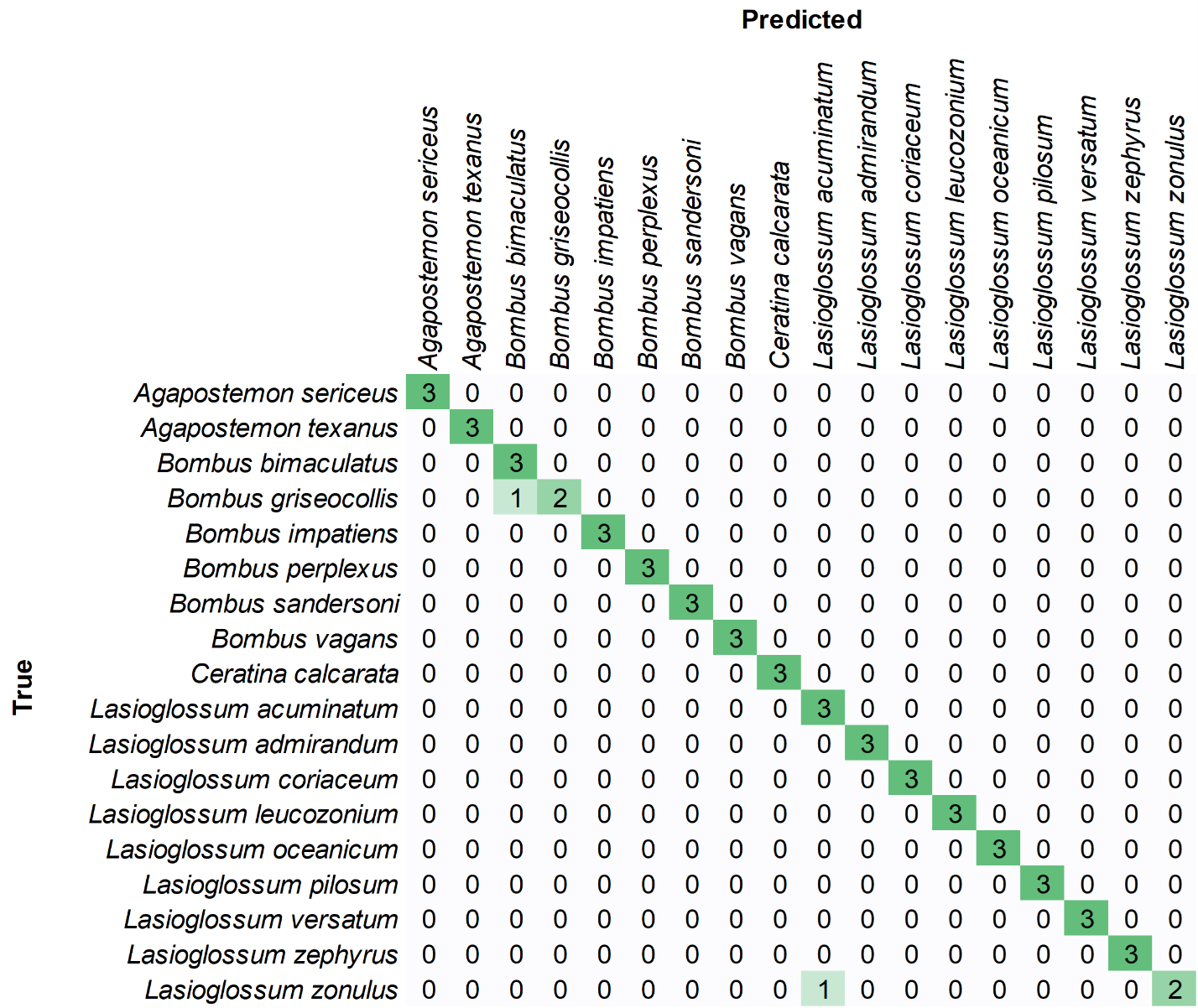


**S7 Fig. Confusion matrix for bee wing image results, test set 2.**


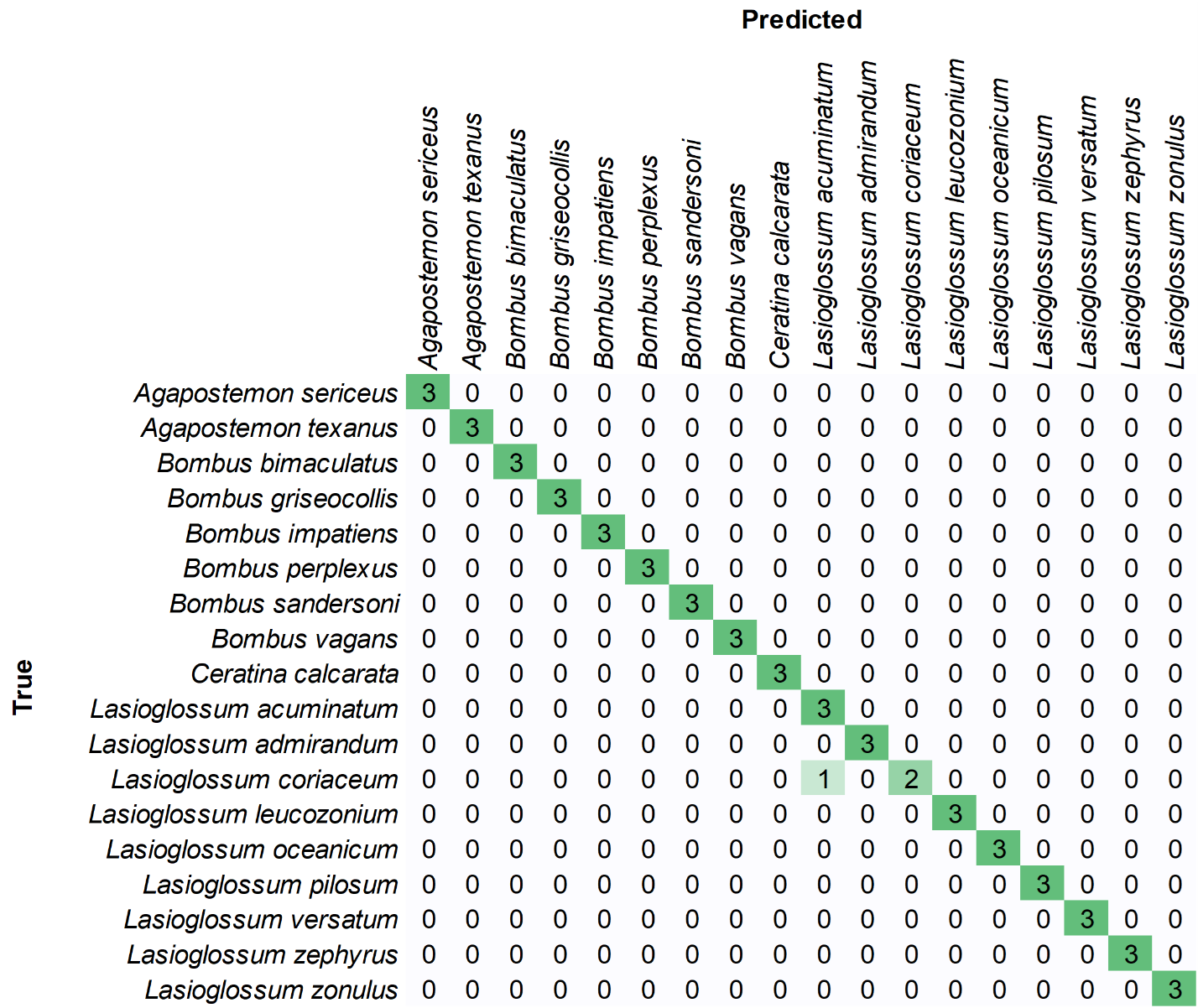


**S8 Fig. Confusion matrix for bee wing image results, test set 3.**
